# Sexual selection contributes to partial restoration of phenotypic robustness in a butterfly

**DOI:** 10.1038/s41598-018-32132-8

**Published:** 2018-09-25

**Authors:** Caroline M. Nieberding, Gilles San Martin, Suzanne Saenko, Cerisse E. Allen, Paul M. Brakefield, Bertanne Visser

**Affiliations:** 1 Evolutionary Ecology and Genetics group, Biodiversity Research Centre, Earth and Life Institute, Université catholique de Louvain, Louvain-la-Neuve, Belgium; 2 Evolutionary Biology Group, Institute of Biology, Leiden University, Leiden, The Netherlands; 3 Division of Biological Sciences, University of Montana, Missoula, MT 59812 USA; 4 Department of Zoology, University Museum of Zoology Cambridge, University of Cambridge, Cambridge, United Kingdom

## Abstract

Phenotypic variation is the raw material for selection that is ubiquitous for most traits in natural populations, yet the processes underlying phenotypic evolution or stasis often remain unclear. Here, we report phenotypic evolution in a mutant line of the butterfly *Bicyclus anynana* after outcrossing with the genetically polymorphic wild type population. The *comet* mutation modifies two phenotypic traits known to be under sexual selection in this butterfly: the dorsal forewing eyespots and the pheromone-producing structures. The original *comet* mutant line was inbred and remained phenotypically stable for at least seven years, but when outcrossed to the wild type population the outcrossed *comet* line surprisingly recovered the wild type phenotype within 8 generations at high (27 °C), but not at low (20 °C), developmental temperatures. Male mating success experiments then revealed that outcrossed *comet* males with the typical *comet* phenotype suffered from lower mating success, while mating success of outcrossed *comet* males resembling wild types was partially restored. We document a fortuitous case where the addition of genetic polymorphism around a spontaneous mutation could have allowed partial restoration of phenotypic robustness. We further argue that sexual selection through mate choice is likely the driving force leading to phenotypic robustness in our system.

## Introduction

Phenotypic variation is the raw material for selection that is ubiquitous for most traits in natural populations. Phenotypic evolution depends partly on underlying genetic variation, on environmental effects, and on the interaction between both factors. There is ample evidence that the amount of phenotypic variation and phenotype evolution are not as large and rapid as could be expected, because phenotypes are often robust to mutations or to environmental perturbations^1,2^. That is, most species maintain abundant genetic variation and experience a wide range of environmental conditions, but phenotypic variation remains relatively low^3–5^. This process has been defined as phenotypic robustness, which refers to individuals of a population that show little phenotypic variation against environmental perturbations or mutations. A critical point to be addressed by research on phenotypic robustness is to determine whether or not robustness evolves under direct selection^1,4,6–9^. Many have argued that robustness can result from non-adaptive processes, such as the developmental architecture underlying traits of interest^9,10^. Others have, however, suggested that robustness can evolve in populations with large population sizes or experiencing high mutation rates in response to stabilizing selection^7,11^. Theoretical work has indeed shown that stabilizing selection reduces phenotypic variation from one generation to the next^12–15^. One theoretical study showed that when sexual selection operates in populations, both stabilizing and directional selection resulting from female mate choice can favor the evolution of robustness^16^. Experimental evidence that (sexual) selection drives the evolution and maintenance of phenotypic robustness is, however, limited (with the exception of work done on RNA viruses)^17–19^.

Mutants have proven to be a valuable resource for understanding evolutionary processes, and here we document a case of phenotypic evolution in a mutant line of the tropical butterfly *Bicyclus anynana* that sheds light on the role of selection and genetic polymorphism in driving phenotypic robustness. Of note, we refer to phenotypic robustness, because the work described here was based on the effects of a single mutation. *B. anynana* has become an important model for studies on developmental plasticity and selection^20^. *B. anynana* shows an extreme form of phenotypic plasticity, seasonal polyphenism, where developmental temperature generates morphologically distinct seasonal forms during the wet and dry African seasons^20^. Dry season individuals that develop at low temperatures are phenotypically cryptic, lacking eyespots on the wings, whereas wet season individuals developing at higher temperature do possess eyespots. Predation on cryptic individuals is less under dry season conditions, whereas eyespots confer an advantage under wet season conditions by deflecting predator attack^21^. The wet and dry phenotypes are produced non-randomly with respect to high or low developmental temperatures at late larval and pupal stages; hence this is likely adaptive phenotypic plasticity. Previous studies revealed that several wing traits play an important role in sexual selection, including the UV-reflecting white pupils of dorsal forewing eyespots^22–24^ and the male sex pheromone (MSP) produced in part by male-specific wing structures called androconia^22,25,26^. Behavioural experiments in which these traits were artificially manipulated in males (i.e. through dissection, covered by nail varnish, or UV-absorbing colours) indeed showed that females exert stabilizing sexual selection on males for round-shaped and small to mid-sized pupils^24^, as well as directional sexual selection on increasing quantities of male sex pheromone components^27,28^.

**Figure 1:**
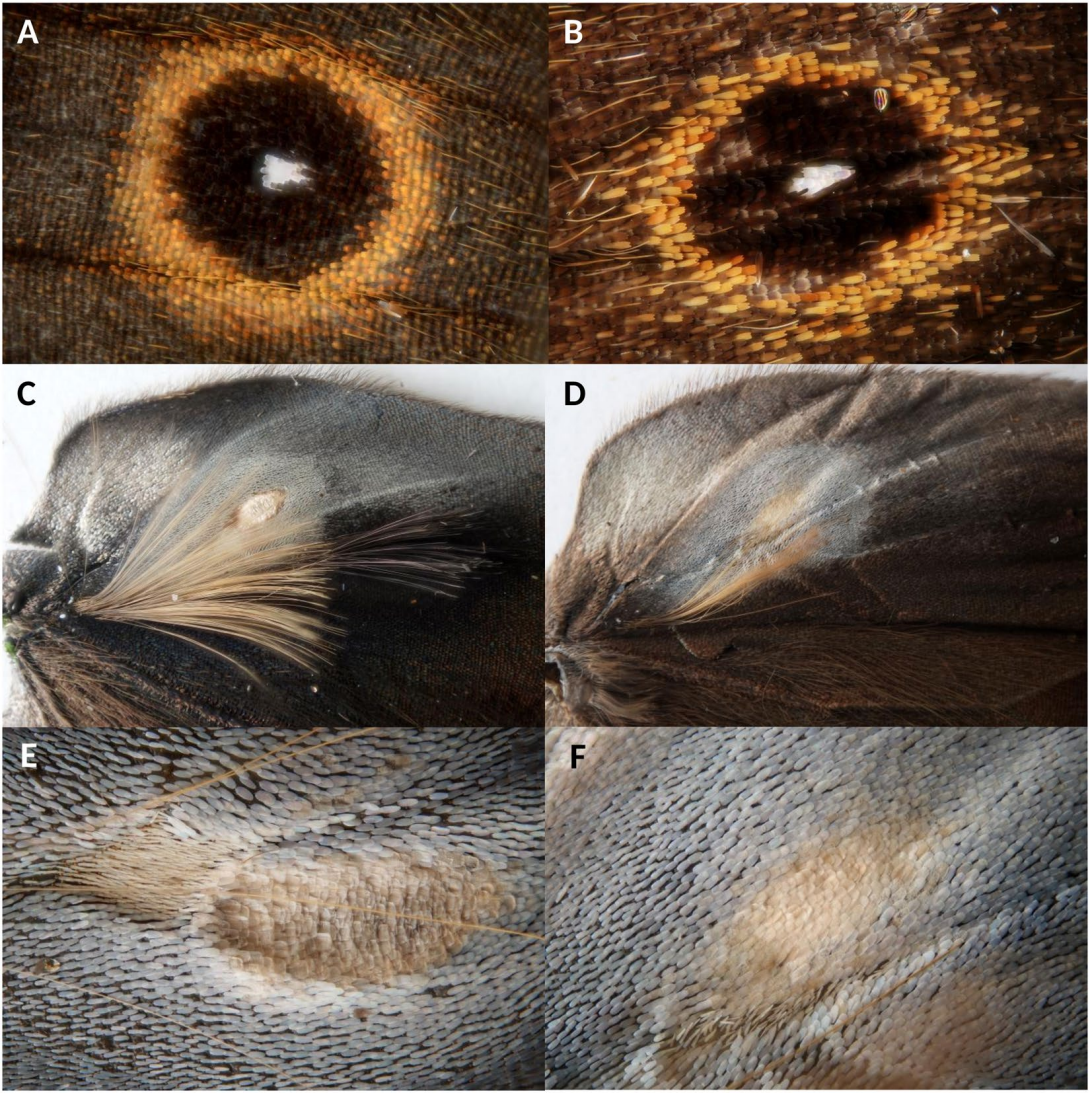
Morphological differences between wild type (left) and *comet* (right) individuals: posterior eyespot on the dorsal side of the forewing (**A**,**B**), androconial first and second hairpencils along with the second androconial patch on the dorsal side of the hindwing (**C**,**D**), and detail of the second androconial patch (after removal of the hair pencils; **E**,**F**).

A *B. anynana* mutant with extensive phenotypic changes on wing traits affecting male mating success appeared sporadically in the wild type laboratory-reared population of *B. anynana*^29–31^. This recessive and pleiotropic mutation, *comet* (*cc*), produces pear-shaped dorsal forewing eyespots (i.e. “comet-shaped”) instead of round eyespots, and the androconia (i.e. pheromone-producing structures) are either reduced in size on the forewing or absent on the hindwing^29,32^ (Fig. 1). As these key secondary sexual traits are affected in inbred *comet*, we inferred that *comet* males would show a reduced mating success compared to wild type males. Whereas developmental temperature leads to seasonal polyphenism in wild types, the *comet* mutant phenotype was stably expressed in the laboratory at various developmental temperatures for at least seven years^29,30,32^.

In this study, we outcrossed the *comet* inbred line to the wild type population that displays high levels of heterozygosity^33^ in order to restore genetic variation throughout the genome typical of the wild type population in individuals with the *comet* mutation. Surprisingly, in the next few generations (i.e. 6–8) we observed that most individuals of the outcrossed *comet* line that were reared at 27 °C degrees did not express the *comet* phenotype and could not be distinguished from wild types. Yet, when reared at 20 °C, the same outcrossed *comet* line again fully expressed the *comet* phenotype. Outcrossing thus resulted in variation in genetic backgrounds, where the mutant phenotype can overlap largely with that of wild types depending on the developmental temperature. In order to document these fortuitous qualitative observations, we quantified the effect of the *comet* mutation in the outcrossed *comet* line on both morphological (eyespot size and shape, androconia presence and size) and physiological (amounts of male sex pheromone components) secondary sexual phenotypic traits by comparing outcrossed *comet* with wild types reared at various temperatures typical of the dry (20 °C) and wet (27 °C) seasonal forms. We used this phenotypic recovery to perform behavioural experiments comparing the mating success of males from the outcrossed *comet* (*cc*) line displaying the *comet* phenotype entirely or not anymore, with wild type (++) males competing for wild type (++) wet seasonal females. Outcrossed *comet* males displaying the *comet* phenotype performed less well compared to outcrossed *comet* males showing the wild type phenotype and wild type males. We propose that phenotypic robustness was partially recovered by epistatic interactions between the *comet* mutation itself and polymorphisms present among the outcrossed genetic backgrounds. We further argue that sexual selection through female preference for round-shaped and small to mid-shaped eyespot pupils and/or for large male sex pheromone quantities has fuelled the rapid recovery of phenotypic robustness in the outcrossed *comet* line once phenotypic variation for sexually-selected traits became available among *comet* males. This could be, to the best of our knowledge, the first indirect evidence that sexual selection can produce phenotypic robustness of sexually selected traits.

## Results

### Effect of the *comet* mutation on male wing secondary sexual traits

**Figure 2:**
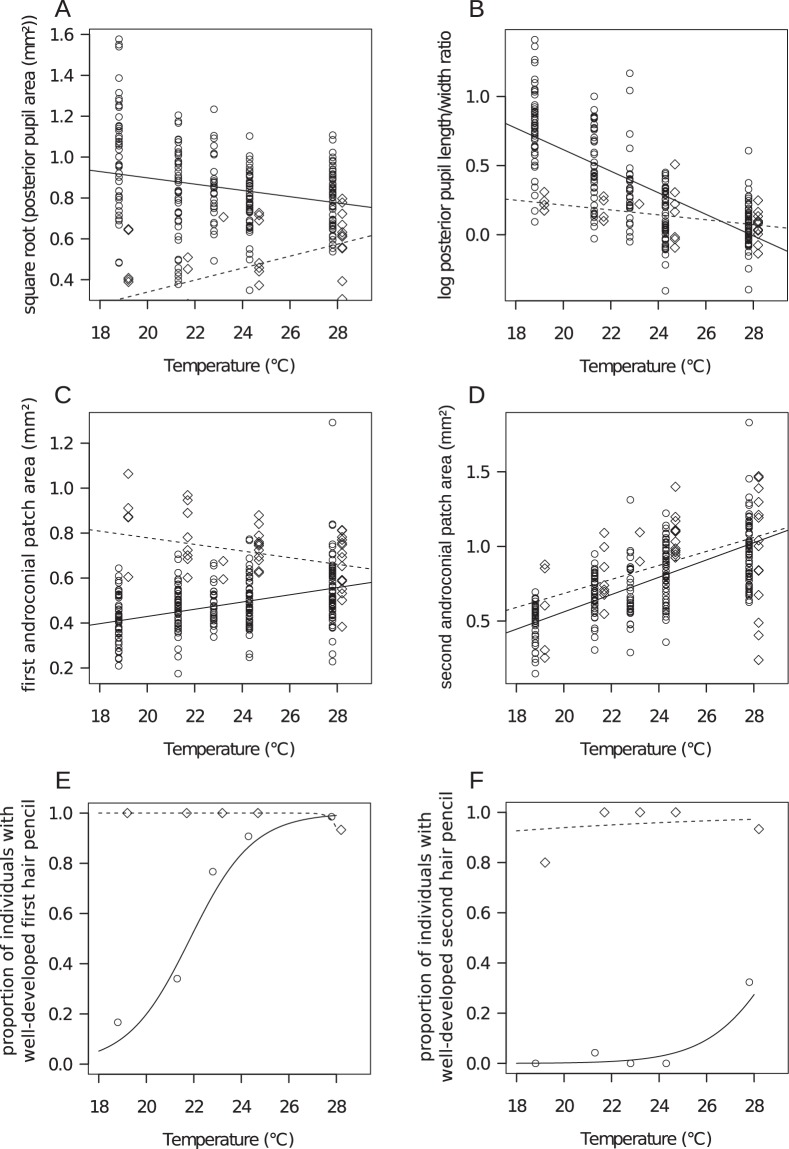
Six male morphological traits were measured for wild type stock (diamonds) and outcrossed *comet* mutants (circles) across 5 breeding temperatures, including (**A**) the posterior pupil area in mm^2^ (square root transformed), (**B**) the posterior pupil length/width ratio (log transformed), (**C**) the first androconial spot area (mm^2^), (**D**) the second androconial spot area (mm^2^), (**E**) the proportion of individuals with a well-developed first hairpencil, and (**F**) the proportion of individuals with a well-developed second hairpencil. The lines represent the corresponding mixed model predictions for wild type (dotted lines) and *comet* (continuous lines) which were corrected for wing size on graphs A, C and D.

Within 6–8 generations following outcrossing, we quantified the phenotypic traits affected by the *comet* mutation by comparing eyespot shape and size, as well as androconia size, between families from the wild type population and the outcrossed *comet* line. When reared at 27 °C, the outcrossed *comet* line had almost completely recovered the wild type phenotype: the eyespot pupil shape was circular compared to the elongated pupils of the original inbred *comet* line, whereas the size of the first androconial spot and presence of the second androconial hairpencil were similar between outcrossed *comet* families and wild type families (Figs 1 and 2). In contrast, phenotypes of outcrossed *cc* families displayed increasing differences compared to the wild type when developmental temperature was decreased (Fig. 2, Table 1): the eyespot pupil shape became more elongated, the first androconial patch was reduced and the second androconial hairpencil faded away with decreasing rearing temperatures. At lower developmental temperatures the phenotype was thus more similar to the inbred *comet* line. Several generations after outcrossing, the phenotypic effects of the *comet* mutation had thus become strongly temperature-dependent for most traits, and the effect of the mutation was uncoupled across the set of six measured traits (Table 1).

**Table 1:** Model estimates (treatment contrasts with the intercept) for 6 male morphological traits involved in sexual selection from wild type and *comet* mutants (type) across 5 breeding temperatures.

Posterior pupil surface (mm², square root transformed)	Estimate	Std.Error	95% credible interval	p
Intercept	0.7756	0.0304	[0.7124; 0.8374]	<0.0001***
forewing surface (mm², centred on the mean)	0.0063	0.0012	[0.0040; 0.0087]	<0.0001***
type (stock)	−0.2017	0.0671	[−0.3379; −0.0676]	0.0058**
temperature (°C, centred on 27 °C)	−0.0154	0.0037	[−0.0228; −0.0080]	<0.0001***
type * temperature	0.0446	0.0105	[0.0239; 0.0656]	<0.0001***
**Posterior pupil length/width (log transformed)**	**Estimate**	**Std.Error**	**95% credible interval**	**p**
Intercept	−0.0084	0.0318	[−0.0726; 0.0558]	0.7806
type (stock)	0.0811	0.0777	[−0.0750; 0.2391]	0.3030
temperature (°C, centred on 27 °C)	−0.0780	0.0046	[−0.0871; −0.0689]	<0.0001***
type * temperature	0.0603	0.0138	[0.0327; 0.0876]	<0.0001***
**First androconial patch surface (mm²)**	**Estimate**	**Std.Error**	**95% credible interval**	**p**
Intercept	0.5576	0.0206	[0.5161; 0.5994]	<0.0001***
hindwing surface (mm², centred on the mean)	0.0032	0.0007	[0.0018; 0.0045]	<0.0001***
type (stock)	0.1037	0.0422	[0.0234; 0.1867]	0.0160*
temperature (°C, centred on 27 °C)	0.0160	0.0022	[0.0117; 0.0204]	<0.0001***
type * temperature	−0.0308	0.0057	[−0.0421; −0.0198]	<0.0001***
**Second androconial patch surface (mm²)**	**Estimate**	**Std.Error**	**95% credible interval**	**p**
Intercept	1.0250	0.0341	[0.9579; 1.0915]	<0.0001***
forewing surface (mm², centred on the mean)	0.0094	0.0011	[0.0073; 0.0115]	<0.0001***
type (stock)	0.0350	0.0692	[−0.1020; 0.1677]	0.5984
temperature (°C, centred on 27 °C)	0.0580	0.0033	[0.0514; 0.0647]	<0.0001***
type * temperature	−0.0111	0.0088	[−0.0289; 0.0057]	0.2050
**Presence of well-developed first hairpencil**	**Estimate**	**Std. Error**	**z value**	**p**
Intercept	4.6489	0.6301	7.3778	<0.0001***
type (stock)	−1.8899	1.2852	−1.4706	0.1414
temperature (°C, centred on 27 °C)	0.7554	0.0968	7.8064	<0.0001***
type * temperature	−4.8537	432.6316	−0.0112	0.9910^+^
**Presence of well-developed second hairpencil**	**Estimate**	**Std. Error**	**z value**	**P**
Intercept	−0.9744	0.3671	−2.6545	0.0079**
type (stock)	4.5342	1.4172	3.1994	0.0014**
temperature (°C, centred on 27 °C)	0.6429	0.1514	4.2467	<0.0001***
type * temperature	−0.54	0.2825	−1.9113	0.0560(*)

The four first models are linear mixed models with family as random effect (not shown) and normal error distribution. The inference on model parameters is based on 10000 MCMC simulations. The two last models (presence/absence of well-developed hairpencils) are generalized linear mixed models with family as random effect (not shown), binomial error distribution and logit link function. The inference on parameters is based on approximate z tests. The temperature values are centred on 27 °C so that type effect estimates the difference between *comet* and wild type at 27 °C. (^+^) The type x temperature interaction was not significant for the first androconial hairpencil (p > 0.99), but this model had some estimation problems due to the high proportion of “presence” in wild type individuals; yet the graphs (Fig. 2 panels E, F) show that the models provide a good fit of the data and that there is no doubt that the differences observed between *comet* and wild type for the androconial hairpencils depend on temperature (i.e. significant interaction) too.

### Effect of the *comet* mutation on male sex pheromone quantities

**Figure 3:**
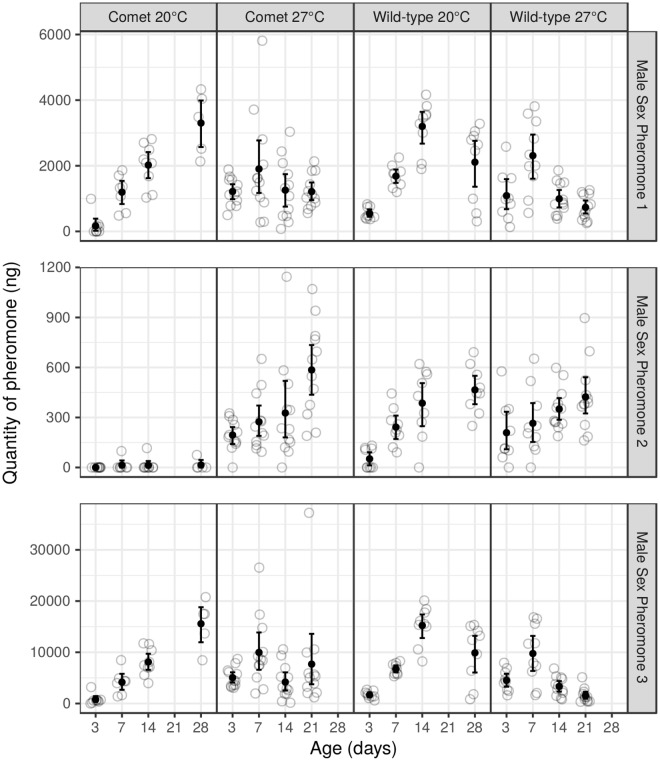
Male sex pheromone (MSP; top = MSP1; centre = MSP2; bottom = MSP3) titres of *comet* (*cc*) and wild type (++) males reared at 20 °C and 27 °C and sampled at 5 different ages (3, 7, 14, 21, 28). At 20 °C, no individuals were sampled at 21 days, while at 27 °C, and no individuals were sampled at 28 days. The open grey circles show the observed values, the black dots represent their mean and the error bars represent bootstrap 95% confidence intervals for the mean. Some random noise has been added on the x axis to limit overplotting.

The quantities of male sex pheromone components were compared between wild type and *comet* males randomly chosen from the outcrossed *comet* line about 6 to 8 generations after outcrossing the *comet* mutant line to test if morphological changes induced by the *comet* mutation affected male sex pheromone production. Male sex pheromone production did not differ between wild type and outcrossed *comet* males reared at a higher temperature, but differed strongly at the lower temperature. At 27 °C, titres of MSP1, MSP2 and MSP3 of wild type and outcrossed *comet* males displayed a similar pattern across age classes (Fig. 3; none of the age x type interactions were significant at the 0.05 level: MSP1: F = 1.1, df = 3, p = 0.35 - MSP2: F = 1.02, df = 3, p = 0.38 - MSP3: F = 1.83, df = 3, p = 0.14). In stark contrast, patterns of MSP titres differed strongly between *comet* and wild type males when butterflies were reared at 20 °C. The production of MSP2 was almost completely suppressed in all ages in outcrossed *comet* males reared at 20 °C, due to absence of the corresponding androconia (Fig. 3; age x type interaction: F = 11.02, df = 3, p < 0.0001) producing this component in the wild type population^25^. Additionally, MSP1 and MSP3 titres of outcrossed *comet* and wild type males both progressively increased, but peaked at 28 days of age in outcrossed *comet* males versus 14 days of age in wild type males. MSP1 and MSP3 titres subsequently decreased in wild type males (age x type interaction MSP1: F = 10.79, df = 3, p = 0.001; MSP3: F = 10.75, df = 3, p = 0.005) (Fig. 3). MSP1 and MSP3 titres at a single age class (14-day old) in outcrossed *comet* males were similar to MSP titres of the younger age class in wild type males (8-day old). Thus the rate of increase of MSP1 and MSP3 titres was slower in outcrossed *comet* than in wild type males at 20 °C.

### Effect of the *comet* phenotype on male mating success

Mating success of males with *comet* or wild type phenotypes was compared during two behavioural experiments estimating male mating success under semi-natural conditions in a large tropical greenhouse. In the first experiment, we competed wild type males to outcrossed *comet* males of the F_3_ generation, most of which (540/600) had reduced androconia and modified eyespot pupils typical for *comet* mutants^32^. Mating success of outcrossed *comet* males (*cc*) was significantly lower than that of heterozygote (*c*+) or wild type males (++) and was similar for all three replicates (both the total and pooled G-tests were significant, as well as the single G-tests for two out of three replicates; Table 2). In a second experiment, only outcrossed *comet* males with circular-shaped eyespot pupils and reduced androconial hairpencils from the F_4_ generation were selected to compete to wild type males for mating. Mating success of outcrossed *comet* (*cc*) males was significantly lower than that of heterozygote (*c*+) or wild type (++) males in the first replicate, but not in the second replicate, with non-significant pooled and global G-tests (Table 2). In both experiments, outcrossed *comet* (*cc*), heterozygote (*c*+) and wild type (++) males were recaptured in similar proportions to those at which they were released (all G-tests were non-significant at the 0.05 level; Table 2); hence male survival was similar among competing groups of males.

**Table 2:** A. Male recapture rates in behavioural experiments and replicated G tests for goodness of fit for each experiment. B. Male mating success in behavioural experiments and replicated G tests for goodness of fit for each experiment.

A. Male recapture rate - First experiment						
++	*c*+	*cc*	*type of G test*	*df*	*G*	*P*
33	18	22		2	4.82	0.0899
14	19	21		2	1.49	0.4742
42	45	33		2	2.00	0.3675
			Total	6	8.31	0.2160
89	82	76	Pooled	2	1.03	0.5983
			Heterogeneity	4	7.29	0.1215
**Male recapture rate - Second experiment**						
++	***c***+	***cc***	***type of G test***	***df***	***G***	***p***
18	21	32		2	0.92	0.6306
16	16	34		2	0.06	0.9701
			Total	4	0.98	0.9124
34	37	66	Pooled	2	0.31	0.8567
			Heterogeneity	2	0.67	0.7141
**B: Male mating success - First experiment**						
***++***	***c***+	***cc***	***type of G test***	***df***	***G***	***p***
23	18	5		2	13.22	0.0013**
12	15	4		2	7.18	0.0276*
14	12	6		2	3.54	0.1706
			Total	6	23.93	0.0005***
49	45	15	Pooled	2	22.02	<0.0001***
			Heterogeneity	4	1.91	0.7527
**Male mating success - Second experiment**						
++	***c***+	***cc***	***type of G test***	***df***	***G***	***p***
13	11	9		2	7.24	0.0268*
5	6	11		2	0.09	0.9555
			Total	4	7.33	0.1193
18	17	20	Pooled	2	4.17	0.1242
			Heterogeneity	2	3.16	0.2059

## Discussion

Our results provide evidence for partial restoration of temperature-dependent canalization of the wild type phenotype through sexual selection. Individuals with the recessive, pleiotropic *comet* mutation deviate from wild types in eyespot size, as well as androconial size and shape, which are of critical importance for male mating success^22,24,25,27^. The *comet* phenotype was originally expressed similarly at all developmental temperatures, but outcrossed *comet* individuals displayed phenotypic plasticity for trait expression in response to temperature and expression of the abnormal sexually-selected traits became uncoupled. At 27 °C outcrossed *comet* males recovered the first androconial hairpencil and the second androconial area, and formed an eyespot similar in shape to that of wild type males. Male sex pheromone quantities at different ages did not differ between outcrossed *comet* males and wild types at 27 °C. At 20 °C outcrossed *comet* males did conserve the abnormal morphological phenotype typical of the original inbred *comet* line and did not produce MSP2, whereas MSP1 and MSP3 were produced with a delay. Behavioural experiments then revealed that outcrossed *comet* males (*cc*) were less attractive than wild type (++) and heterozygote (*c*+) males (i.e. displaying the wild type phenotype, as *comet* is a recessive mutation) under semi-natural conditions. This is in line with previous findings in *B. anynana*, which showed that females prefer round mid-sized eyespots^22,23^ and males producing specific amounts of male sex pheromones^22,25,27,28^. Outcrossing the *comet* mutation thus resulted in a decanalization of the phenotype, likely as a result of epistatic interactions of the *comet* mutation with the added genetic polymorphism from wild types; sexual selection thus acted to push the population back towards the wild type state.

### Temperature-dependent expression, seasonal dimorphism and regulation

Restoration of phenotypic robustness in outcrossed *comet* depended on developmental temperatures, i.e. outcrossed *comet* males appeared more like wild types only at the higher temperature (27 °C). *B. anynana* is a species showing a well-developed temperature-sensitive dimorphism, i.e. wet and dry seasonal forms; hence developmental pathways are in place to mediate temperature-dependent expression of the phenotype. Polyphenism in *B. anynana* is induced by developmental temperature in the late larval stage (5^th^ instar larvae and early pupal stage)^34,35^, and affects a wide range of morphological^23,36^, physiological^27,37^, and behavioural traits^34,38,39^, including secondary sexual traits. Differentiation into distinct seasonal forms results from hormone signalling, specifically increased ecdysteroid levels during early pupal stages^40,41^. Several key genes have been implicated in the regulation of eyespot number and size, including Distal-less, Engrailed, and Spalt, where the former was shown to regulate focal differentiation and eyespot signalling, as well as scale melanization^42^. A recent study revealed that low versus high developmental temperatures indeed induce highly differential transcriptional responses, suggesting a genome-wide genetic architecture underlies each temperature-dependent developmental program^43^. No genetic variation for phenotypic plasticity was found in the lab-reared sampled population originating from Malawi (i.e. the same population as was used here), however, likely as a result of purifying selection favouring distinct seasonal forms in the highly predictable natural tropical environment of *B. anynana*^43^. Phenotypic robustness in our outbred *comet* line was thus likely triggered by activation of the genetic and developmental pathways underlying the wet seasonal wild type form only. Interestingly, similar temperature-dependent responses are often found in *Drosophila melanogaster* wing mutant lines, such as *vestigial*, where normal wing phenotypes are only restored at higher temperatures^3,44,45^.

### The role of genetic evolution, polymorphism and epistasis in phenotypic robustness

The original *comet* line went through a major bottleneck, was inbred, and showed an abnormal, though stable, phenotype at a range of developmental temperatures for at least 7 years following isolation of the mutant from the wild type population^29,30,32^. Outcrossing of the inbred *comet* line to a large number of wild type individuals led to fading of the *comet* phenotype within a few generations, but only at the high developmental temperature. Genetic evolution by natural or purifying selection, where a disadvantageous mutation would be removed from the population, might seem as an intuitive explanation for recovery of wild type phenotypes in outcrossed *comet* males. Alternatively, genetic drift, i.e. random changes in allele frequencies, might explain the disappearance of *comet* phenotypes following outcrossing. Natural selection pressures due to predation, parasites, food limitation or mating barriers are absent in our laboratory rearing environment, however, suggesting that forces other than natural selection acted on the *comet* outcrossed line. Moreover, we observed that phenotypes converged back to the original wild type values. If drift were indeed at play, it seems highly unlikely that random changes would coincidentally lead to phenotypic values that are identical to wild type phenotypes. Natural selection nor drift were thus involved in the recovery of wild type phenotypes. Moreover, both processes of genetic evolution would imply that outcrossed *comet* males lost the original *comet* mutation. We cannot confirm the presence of the *comet* mutation by sequencing or other molecular methods, because the *comet* mutation has not yet been located, despite efforts of others to do so^31^. Our observations, however, directly contradict this possibility. *Comet* shows a Mendelian pattern of inheritance and only homozygous recessive F_2_ individuals (i.e. a quarter of all offspring produced by crossing the F_1_ amongst itself) were used to set up the outcrossed *comet* line for all experiments described here. The abnormal *comet* phenotype was further found to be expressed in successive generations of the outcrossed *comet* line in individuals reared at a low development temperature, but not when reared at a higher temperature, and was expressed increasingly with increasing temperature in our family-design experiments (Fig. 2). Trait- and temperature-specific phenotypic recovery in the outcrossed *comet* line thus excludes the possibility that the *comet* mutation was simply lost.

Morphological trait values showed a much higher variance at the lower temperature in outbred *comet* males compared to wild types. Addition of genetic polymorphism thus decanalised the abnormal *comet* secondary sexual phenotypic traits in the *comet* outbred line. The addition of genetic polymorphism thus serves as a mechanism by which variation was reintroduced and phenotypic robustness restored over subsequent generations. Other work has shown that robustness to mutations in the P450 protein was higher in larger and more polymorphic populations compared to smaller and less polymorphic populations such that genetic polymorphism is likely responsible for higher robustness^46^. It was further revealed that the genetic background in which the HSP90 chaperone is expressed can have a large effect on resultant phenotypes, as is the case in *Drosophila*^47^. How would genetic polymorphism restore phenotypic robustness of the *comet* line? Most mutations are background-dependent (i.e. show epistatic effects) and variation accumulated in the wild type population caused diversification of genetic backgrounds with which the *comet* mutation interacted epistatically. Some genetic backgrounds of the wild type population produced a particular phenotypic effect with the *comet* mutation and others did not. The diverse genetic background of the wild type population would then lead the *comet* outcrossed line as a whole to express more novel phenotypes compared to the *comet* inbred line^9,48,49^. This conceptual argument positively correlates robustness with evolvability, which has been formalized in mathematical models of so-called neutral networks in genotype space (more recently termed genotype networks) and has some empirical support^8,9,19,50^. In our *comet* case study, genetic polymorphism present in the wild type population was not sufficient to *maintain* phenotypic robustness, otherwise the *comet* mutant would not have appeared in the wild type population in the first place. Genetic polymorphism thus merely allowed partial *restoration* of phenotypic robustness after polymorphism was added to the original *comet* inbred line.

### Sexual selection as a driving force for restoration of phenotypic robustness

Replicated mating success experiments with outcrossed *comet* males that partially recovered wild type phenotypes overall revealed that outcrossed *comet* males with ‘less abnormal’ (i.e. closer to the wild type) phenotypes had higher mating success than males with more abnormal phenotypes (i.e. with more pronounced changes in eyespot and androconial traits). Decreased mating success of these outcrossed *comet* males may be due to their larger (compared to wild type) eyespot pupils^24^, and reduced male sex pheromone transfer to female antenna during courtship as a consequence of the reduced second hairpencil and androconial spots^25^. The amount of male sex pheromone components present on male wings are indeed correlated with the size of the androconial areas^27^. Females exert stabilizing selection on eyespot size and shape^24^ and directional selection on male sex pheromone components^27^ through mate choice decisions. Females thus preferred males that appeared to be (more) like wild types. As the *comet* mutation affects secondary sexual traits that we know are under sexual selection in wild type *B. anynana*, we suggest that sexual selection against the *comet* phenotype is the driving force that led to the restoration of phenotypic robustness against the mutation. This conclusion lines up well with a first modelling study by Fierst^16^ who suggested that female mate preferences increase male phenotypic robustness under three different sexual selection scenarios compared to a randomly mating population. Her theoretical results imply that female choice leads to selection pressures that affect robustness, which thus has the potential to develop in any population experiencing sexual selection^16^. Our results suggest that sexual selection restored phenotypic robustness against the spontaneous *comet* mutation within a few generations at a high rearing temperature, likely by stabilizing selection for *comet* phenotypic variants that were closer and closer to wild type trait values.

A role for sexual selection in accelerating adaptation through purging of deleterious mutations and decreasing the mutational load has been suggested previously, but was mainly based on indirect evidence^51,52^. Furthermore, scant direct evidence was obtained through measurements of sexual selection against a single deleterious allele, where sexual selection increased purifying selection^53–55^. A major assumption in these studies was that these deleterious alleles affected non-sexual traits and overall condition, leading to high variance in male mating success and purging through intense male-male competition^52^. Under that scenario, a male performs less well when in competition, not because a deleterious mutation affects the secondary sexual trait that is under selection directly, but because his overall health and performance (including mating success and courtship persistence) is negatively affected. Moreover, conflicting results revealed an opposite effect of sexual selection on purging of the mutational load^56^. Unlike other studies, the *comet* mutation directly affects several key secondary sexual traits that are under both stabilizing and directional sexual selection; hence we provide more direct evidence of the role played by sexual selection in purging male phenotypes that are suboptimal regarding female mate preference.

### Prospects for future research

One of the most exciting findings of this study is that sexual selection contributes to the restoration of phenotypic robustness. We should note, however, that we tested sexual preferences of wild type females, and not of *comet* females, where we assumed that both would have similar preferences for male traits. This may not be true, because, for example, learning through self, oblique or horizontal imprinting of surrounding phenotypes during sexual maturation is known to affect female sexual preferences in insects, also in *B. anynana*^57^. *Comet* females may thus learn to prefer *comet*-looking males because they imprinted on each other in our group-rearing system before experiments were performed, leading to assortative mating (reviewed by Dion, Monteiro, Nieberding, in review). Learning is biased in *B. anynana*, however, in that females can learn to prefer supra-natural sexual stimuli, but not reduced wing ornamentation; females may, therefore, not be able to learn to prefer drab *comet* males^57^. Follow-up experiments should, however, take into account that sexual preferences and mate-choice decisions of wild type and *comet* females may differ.

The role of sexual selection on canalizing phenotypic robustness in *comet* mutants could be strengthened by two additional experiments: first, experimental evolution comparing a treatment with and without (monogamous) sexual selection could show that the *comet* phenotype disappears in the outcrossed *comet* line despite still being fixed for the *cc* mutation only when sexual selection is present (e.g.^53^). Second, the mating success of the outcrossed *comet* line could have been compared between *cc* males with high expressivity versus *cc* males with lower expressivity of the *comet* phenotype, to show that mating success of the later was higher than that of the former.

The role of genetic polymorphism and epistatic interactions in decanalizing the *comet* phenotype needs to be tested more rigorously. To restore genetic polymorphism around the *comet* mutation, 50 *comet* individuals were randomly mated with 100 wild types, generating an outcrossed *comet* F_1_ that was subsequently crossed among itself to produce the F_2_. This population-level approach was based on a single cross with wild types; hence a single replicate was used. This could lead to confounding effects regarding: *1)* the role of inbreeding in the outbred *cc* population compared to wild types; *2)* the role of one, few or multiple polymorphisms contributing to the phenotypic variability observed in the outbred *comet* line. First, genetic variability in the outbred *comet* line was about half that of the outbred wild type population, i.e. half of the chromosomes of the F_1_ are derived from the inbred *comet* line. Inbreeding is known to affect the general condition and mating success of individuals including in *B. anynana*^28,58^, likely through effects on flight ability^58^. Our mating experiments were performed under similar semi-natural conditions as in^58^; hence outbred *comet* males may indeed have suffered from lower courtship activity reducing mating success, in addition to abnormal sexual traits. However, outbred *comet* males more similar in sexual trait values to wild types also had higher mating success (i.e. second mating success experiment), suggesting a sufficient level of outbreeding was attained to lead to comparable fitness effects. Also, inbreeding in the outbred *comet* line was likely alleviated by inbreeding depression during generations the outcrossed *comet* line was maintained before experiments were performed: inbred *comet* individuals would thus have a lower fitness compared to more outbred *comet* individuals, increasing the average population outbreeding level for generations following our initial cross. The composition of the male sex pheromone was indeed found to dictate female mate preferences in *B. anynana* when inbred vs outbred males were compared^28^. It is thus likely that inbreeding depression did occur prior to experiments in our rearing cages, likely through olfactory communication on inbreeding status.

Second, the *comet* mutation remained stable in our lab at least 7 years, and our results suggest that the addition of genetic polymorphism led to partial recovery of phenotypic robustness against the deleterious phenotypic effects of the *comet* mutation in outcrossed *comet* mutants. It remains unclear, however, how many genetic polymorphisms from the wild type stock interacted epistatically with the *comet* mutation to decanalize the comet phenotype. Modifier genes have long been known to affect phenotypic expression of mutations^59^. A prime example is the *vestigial* mutant in *Drosophila* (affecting wing and haltere development), where several modifier genes, i.e. additional loci, epistatically modify the phenotypic effect of *vestigial*^60–63^. Crossing a laboratory *vestigial* line to either field-caught or laboratory wild type individuals indeed restored the wild type phenotype within 20 to 40 generations, respectively (despite the continued presence of *vestigial* in the genome)^44^. The faster recovery when crosses were done with field-caught individuals and the substantial number of generations needed for phenotypic recovery (i.e. >20 generations) suggests that multiple loci act as modifiers here. In the case of *comet*, our observations could have resulted from the introduction of a variant that interacts epistatically with *comet* and restores the wild type phenotype even in homozygous state. This variant could then increase in frequency over consecutive generations through sexual selection. This idea is further substantiated by the rate with which phenotypic restoration occurred, i.e. within a few generations, suggesting few genes were acting as modifiers on the *comet* phenotype. To reject the role of a single or few polymorphisms, as opposed to wild type overall genetic polymorphism as being responsible for epistatic restoration of phenotypic robustness, we propose an experimental set-up in which multiple replicates of single pair-matings between one inbred *comet* and one wild type *B. anynana* individual would be performed. If all pair crosses then lead to phenotypic restoration in the F_2_ outbred *comet* families, genetic polymorphism and epistasis unequivocally play a critical role in the restoration of phenotypic robustness in *B. anynana*.

## Material and Methods

### Insects

An outbred wild type population of the African butterfly, *Bicyclus anynana* (Lepidoptera: Nymphalidae), was established in 1988 from over 80 gravid females collected from a single source population in Malawi, Africa. *B. anynana* larvae were maintained on a maize-based diet (*Zea mays*), whereas adults were fed mashed banana (*Musa acuminata*). High levels of heterozygosity were maintained by using laboratory population sizes that ranged between 400 and 600 adults per generation^30,33^. The wild type population was reared in climate rooms at a set of different temperature (20–27 °C) and humidity regimes (60 to 80% RH) that represent the natural range of environmental variation present in the field. The two extreme temperatures, 20 °C (±1 °C) and 27 °C (±1 °C), represent the developmental temperature typical of the dry and wet seasonal forms under laboratory conditions, respectively.

### The *comet* mutant phenotype

*Comet* is a spontaneous, recessive and pleiotropic mutation that arose in a single individual of the *B. anynana* wild type population before 1998^29,31,32^. The *comet* line was founded by homozygous “*cc”* individuals displaying pear-shaped (“comet-shaped”) instead of round eyespots on the dorsal and ventral sides of fore- and hind-wings^24^ following a cross with a wild type individual. Genetic diversity within the *comet* line is expected to be low, first due to the initial bottleneck as this spontaneous recessive mutation occurs very rarely in the wild type population, and second because the *comet* line was subsequently kept in the laboratory at a relatively small population size for years (~30 to 100 individuals per generation). The *comet* mutant line stably displayed an abnormal phenotype in the laboratory for at least seven years, while reared at various developmental temperatures^30,32,64^.

### Experimental crossings

The *comet* inbred line (~50 individuals) was outcrossed to a large number of wild type individuals (~100 individuals) that displays high levels of heterozygosity^33^ in order to partly restore the genetic polymorphism of the wild type population around the *comet* mutation. Specifically, virgin *comet* females were crossed with virgin wild type males in one cage, and virgin *comet* males were crossed with virgin wild type females in another cage, and the eggs collected in both cages were mixed and reared together to produce the F_1_ generation (*c*+). The collected F_1_ generation (*c*+) displayed a wild type phenotype and was crossed among itself to produce a F_2_ generation in which ¼ of the individuals displayed the *comet* phenotype and were “*cc*”, similar to findings in Beldade *et al*.^31^. These F_2_ *comet* “*cc”* individuals were then selected to produce the next generations of what we call hereafter the “outcrossed *comet* line”. The outcrossed *comet* line was kept at 20 °C when not used for collecting eggs or for experiments and all experiments were produced from eggs collected from this single, genetically mixed, line.

### Effect of the *comet* mutation on male wing secondary sexual traits

To quantify the phenotypic effect of the *comet* mutation and assess the effect of developmental temperature on its expression, we reared 3 wild type and 8 *comet* families obtained from eggs collected in the outcrossed *comet* line about 6 to 8 generations after the F_2_ generation at 5 temperatures: 19, 21.5, 23, 24.5 and 27 °C. Eggs were collected from the outcrossed *cc* line and from the wild type population. We measured the following male traits: (i) pupil length/width ratio of the dorsal forewing posterior eyespot pupil (measured as the maximal length of the pupil parallel to the wing vein and the width as the maximum width perpendicular to the length), (ii) pupil area of the dorsal forewing posterior eyespot pupil (approximated from the area of an ellipse with pupil length as major axis and pupil width as minor axis), (iii) the area of the first androconial patch located on the forewing ventral side, (iv) the area of the second androconial patch located on the hindwing dorsal side, (v) the presence/absence of a well-developed hairpencil (functionally associated with the forewing androconia), and (vi) presence/absence of a well-developed hairpencil (associated with the hindwing androconia). Hairpencils were considered to be well-developed when at least 10 hairs were present. These six morphological traits are either directly or indirectly (i.e. androconia size) involved in sexual selection^27,65^. We also estimated the area of the forewing and hindwing by measuring the area between 4 landmarks on each wing. For all morphometric measurements, we recorded the x y coordinates of different landmarks by projecting an image of each morphological structure of interest from a stereomicroscope equipped with a camera lucida onto a graphical tablet. The x y coordinates were then converted into areas or lengths taking into account the magnification and the number of pixels between the coordinates.

### Effect of the *comet* mutation on male sex pheromone quantities

Eggs were collected from the outcrossed *comet* line (*cc*) 6 to 8 generations after the F_2_, and from the wild type population. Individuals were kept at 20 °C or 27 °C throughout development and adult life. Virgin males were sampled for determining male sex pheromone (MSP) quantities at ages 3, 7, 14 and 21 days for individuals kept at 27 °C and ages 3, 7, 14 and 28 days for individuals kept at 20 °C. MSPs were extracted and quantified as described previously^25^. Briefly, one forewing and hindwing per individual were soaked during 5 minutes in 600 µl of hexane, after which 1 ng/µl of internal standard (palmitic acid) was added. Extracts were then analysed on a Hewlett-Packard 6890 series II gas chromatograph (GC) equipped with flame-ionization detector and interfaced with a HP-6890 series integrator with nitrogen as carrier gas. The injector temperature was set at 240 °C and the detector temperature at 250 °C. A HP-1 column was used and temperature increased from the initial temperature of 50 °C by 15 °C/min up to a final temperature of 295 °C, which was maintained for 6 min.

### Effect of the *comet* phenotype on male mating success

To test for behavioural effects of the *comet* mutation on male mating success, we performed behavioural experiments competing wild type (++), heterozygote (*c*+) and outcrossed *comet* (*cc*) males for mating success. Two behavioural experiments were performed that aimed at comparing mating success of wild type males and *comet* males that showed both abnormal pupil shapes and lacked androconia (experiment 1), or *comet* males that had normal pupil shapes but lacked androconia (experiment 2). Specifically, for experiment 1: wild type males were obtained from eggs of the wild type stock population; *comet* males were obtained from the outcrossed *comet* line (F_3_ generation); heterozygote males (*c*+) by crossing 32 F_2_ *cc* virgin females from the outcrossed *comet* line with 30 wild type males, and 30 F_2_ *cc* males from the outcrossed *comet* line with 28 virgin wild type females, in two separate cages. Eggs of the three treatments (*cc*, *c*+ and ++) were collected for 10 days and reared mostly at 27 °C, although eggs from replicates 2 and 3 of experiment 1 were kept at the beginning of their development at 20 °C in order to delay emergence of the adults. This did not affect the production of wet seasonal individuals, as developmental temperature during late larval and early pupal stages determines the adult seasonal phenotype^35^.

We noted that 10% of *cc* males (60 out of 600 males) in the outcrossed *comet* F_3_ generation displayed wild type eyespots, while androconia remained typically “*comet*-like” with the second set of hairpencils being reduced. To test how reduced androconia alone (with normal eyespots) affected male mating success, we crossed these 60 *comet* F_3_ males with 50 *comet* F_3_ females that also had more rounded eyespots to produce the F_4_ generation of the *comet* outcrossed line, which were used in experiment 2. The F_4_ generation of the outcrossed *comet* line produced mostly males with a wild type eyespot shape but *comet*-like reduced androconia. We compared the mating success of these F_4_ outcrossed *comet* males with that of male heterozygote (*c*+) and wild type (++) males obtained as described above for experiment 1.

In both behavioural experiments, groups of 3 to 10-day old virgin males were released in a spacious tropical greenhouse that provided a semi-natural environment for *B. anynana*. Male genitalia were dusted with coloured fluorescent powder^25,58^. In experiment 1, males (*cc, c*+ and ++) competed for matings at a 1:1:1 ratio, with group numbers ranging from 60 to 75 males per group. In experiment 2, wild type (++), heterozygote (*c*+) and *comet* (*cc*) males were released in a proportion of 1:1:2 to mimic an environment in which the wild type phenotype (represented by both ++ and *c*+ males) was as abundant as the *comet* phenotype, with numbers ranging between 25 to 60 males per group. In both experiments, 3 to 10-day old virgin wild type females (50 to 130 per replicate) were released the following morning, to obtain approximately a 2:1 male:female ratio. Males competed for matings during 72 h, after which females were inspected under ultraviolet illumination for fluorescent dust transferred during mating to assess female mate choice. Double matings occurred occasionally (approximately 1 in every 20 matings) and were scored as 1:1. Experiment 1 was repeated three times, and experiment 2 was repeated twice.

### Statistics

All statistical analyses were performed with R 2.12.0^66^, using the lme4 package^67^. To test for effects of *comet* on wing morphology, we used mixed models with family as a random variable, and type (outcrossed *comet* or wild type), temperature (as continuous variable) and their interaction as fixed explanatory variables. We used a normal error distribution for the continuous variables androconial patch area and eyespot pupil size, and a binomial distribution for the hairpencils, which were scored as present or absent. Eyespot pupil ratio data was log transformed and pupil surface was square root transformed to improve homoscedasticity and normality of residuals. For model parameter inference, we used Markov Chain Monte-Carlo simulations (i.e. the mcmc function from the lme4 package) for normal models and approximate z tests for binomial models. Temperature values were centred on the maximum value (27 °C). The “type effect” parameter, therefore, corresponds to the difference between *comet* and wild type at 27 °C. For pupal and androconial patch size, wing area (centred on the mean) was also added as an explanatory variable to control for wing size.

To analyse sex pheromone quantities, individuals reared at 20 °C and 27 °C were analysed separately, because selected age classes differed between the two temperatures. We used linear models with MSP titres as dependent variables and age, type (outcrossed *comet* or wild type) and their interaction as explanatory variables. These explanatory variables were tested with type II F tests (nested models comparison, with main effects tested after removing their interaction from the full model).

To analyse effects of the *comet* mutation on male mating success, replicated G tests of goodness of fit were used as described by Sokal & Rohlf ^68^. A single G test of goodness of fit was computed for each replicate independently and three additional G statistics were calculated: a heterogeneity G test to test whether the different replicates show the same trend, a pooled G test based on the pooled dataset for all replicates and a total G test based on the sum of the single G statistics produced for each replicate.

### Preprint information

This paper was posted on bioRxiv prior to publication^69^.

## Electronic supplementary material

Morphological measurements

Morphological measurements mean repeatability

Pheromone quantities

## Data Availability

All data generated or analysed during this study are included in this published article (and its Supplementary Information files).

## References

[1] Masel J, Siegal ML (2009). Robustness: mechanisms and consequences. Trends Genet..

[2] Masel J, Trotter MV (2010). Robustness and evolvability. Trends Genet..

[3] Waddington CH (1942). Canalization of development and the inheritance of acquired characters. Nature.

[4] Waddington, C. H. *The strategy of the genes*. (Allen and Unwin, 1957).

[5] Félix M-A, Barkoulas M (2015). Pervasive robustness in biological systems. Nat. Rev. Genet..

[6] Meiklejohn CD, Hartl DL (2002). A single mode of canalization. Trends Ecol. Evol..

[7] Wagner GP, Booth G, Bagheri-Chaichian H (1997). A population genetic theory of canalization. Evolution (N. Y)..

[8] Lauring AS, Frydman J, Andino R (2013). The role of mutational robustness in RNA virus evolution. Nat. Rev. Microbiol..

[9] Siegal ML, Leu J (2014). On the nature and evolutionary impact of phenotypic robustness mechanisms. Annu. Rev. Ecol. Evol. Syst..

[10] Flatt T (2005). The evolutionary genetics of canalization. Q. Rev. Biol..

[11] Wilke CO, Wang JL, Ofria C, Lenski RE, Adami C (2001). Evolution of digital organisms at high mutation rates leads to survival of the flattest. Nature.

[12] Lande R (1980). Genetic variation and phenotypic evolution during allopatric speciation. Am. Nat..

[13] Layzer D (1980). Genetic variation and progressive evolution. Am. Nat..

[14] Rice SH (1998). The evolution of canalization and the breaking of Von Baer’s laws: Modeling the evolution of development with epistasis. Evolution (N. Y)..

[15] Kawecki TJ (2000). The evolution of genetic canalization under fluctuating selection. Evolution (N. Y)..

[16] Fierst JL (2013). Female mating preferences determine system-level evolution in a gene network model. Genetica.

[17] Montville R, Froissart R, Remold SK, Tenaillon O, Turner PE (2005). Evolution of mutational robustness in an RNA virus. PLoS Biol..

[18] Sanjuán R, Cuevas JM, Furió V, Holmes EC, Moya A (2007). Selection for robustness in mutagenized RNA viruses. PLoS Genet..

[19] McBride RC, Ogbunugafor CB, Turner PE (2008). Robustness promotes evolvability of thermotolerance in an RNA virus. BMC Evol. Biol..

[20] Brakefield PM, Beldade P, Zwaan BJ (2009). The African butterfly *Bicyclus anynana*: A model for evolutionary genetics and evolutionary developmental biology. Cold Spring Harb. Protoc..

[21] Lyytinen A, Brakefield PM, Lindstrom L, Mappes J (2004). Does predation maintain eyespot plasticity in *Bicyclus anynana*?. Proc. R. Soc. B Biol. Sci..

[22] Costanzo K, Monteiro A (2007). The use of chemical and visual cues in female choice in the butterfly *Bicyclus anynana*. Proc. R. Soc. B Biol. Sci..

[23] Prudic KL, Jeon C, Cao H, Monteiro A (2011). Developmental plasticity in sexual roles of butterfly species drives mutual sexual ornamentation. Science (80-.)..

[24] Robertson KA, Monteiro A (2005). Female *Bicyclus anynana* butterflies choose males on the basis of their dorsal UV-reflective eyespot pupils. Proc. R. Soc. B Biol. Sci..

[25] Nieberding CM (2008). The male sex pheromone of the butterfly Bicyclus anynana: Towards an evolutionary analysis. PLoS One.

[26] San Martin G, Bacquet P, Nieberding CM (2011). Mate choice and sexual selection in a model butterfly species, *Bicyclus anynana*: State of the art. Proceedings of Netherlands Entomological Society.

[27] Nieberding CM (2012). Cracking the olfactory code of a butterfly: The scent of ageing. Ecol. Lett..

[28] van Bergen E, Brakefield PM, Heuskin S, Zwaan BJ, Nieberding CM (2013). The scent of inbreeding: a male sex pheromone betrays inbred males. Proc. R. Soc. B Biol. Sci..

[29] Brakefield PM (1998). The evolution–development interface and advances with the eyespot patterns of *Bicyclus* butterflies. Heredity (Edinb)..

[30] Brakefield PM (2001). Structure of a character and the evolution of butterfly eyespot patterns. Journal of Experimental Zoology.

[31] Beldade P, Saenko SV, Pul N, Long AD (2009). A gene-based linkage map for Bicyclus anynana butterflies allows for a comprehensive analysis of synteny with the lepidopteran reference genome. PLoS Genet..

[32] Brakefield PM, French V (1999). Butterfly wings: the evolution of development of colour patterns. BioEssays.

[33] Van’t Hof AE (2005). Characterization of 28 microsatellite loci for the butterfly *Bicyclus anynana*. Mol. Ecol. Notes.

[34] Bear A, Monteiro A (2013). Male courtship rate plasticity in the butterfly Bicyclus anynana is controlled by temperature experienced during the pupal and adult stages. PLoS One.

[35] Kooi RE, Brakefield PM (1999). The critical period for wing pattern induction in the polyphenic tropical butterfly Bicyclus anynana (Satyrinae). J. Insect Physiol..

[36] Sekimura Toshio, Nijhout H. Frederik (2017). Diversity and Evolution of Butterfly Wing Patterns.

[37] Dion E, Monteiro A, Yew JY (2016). Phenotypic plasticity in sex pheromone production in *Bicyclus anynana* butterflies. Sci. Rep..

[38] Westerman E, Monteiro A (2016). Rearing temperature influences adult response to changes in mating status. PLoS One.

[39] Ng SY, Bhardwaj S, Monteiro A (2017). Males become choosier in response to manipulations of female wing ornaments in dry season Bicyclus anynana butterflies. J. Insect Sci..

[40] Koch PB, Brakefield PM, Kesbeke F (1996). Ecdysteroids control eyespot size and wing color pattern in the polyphenic butterfly *Bicyclus anynana* (Lepidoptera: Satyridae). J. Insect Physiol..

[41] Oostra V (2011). Translating environmental gradients into discontinuous reaction norms via hormone signalling in a polyphenic butterfly. Proc. R. Soc. B Biol. Sci..

[42] Monteiro A (2013). Distal-less regulates eyespot patterns and melanization in *Bicyclus* butterflies. J. Exp. Zool. Part B Mol. Dev. Evol..

[43] Oostra V, Saastamoinen M, Zwaan BJ, Wheat CW (2018). Strong phenotypic plasticity limits potential for evolutionary responses to climate change. Nat. Commun..

[44] Cavicchi S (1989). Developmental effects of modifiers of the vg mutant in Drosophila melanogaster. Dev. Genet..

[45] Waddington CH (1953). Genetic assimilation of an acquired character. Evolution (N. Y)..

[46] Bloom JD, Romero PA, Lu Z, Arnold FH (2007). Neutral genetic drift can alter promiscuous protein functions, potentially aiding functional evolution. Biol. Direct.

[47] Rutherford SL, Lindquist S (1998). Hsp90 as a capacitor for morphological evolution. Nature.

[48] Wagner A (2011). The molecular origins of evolutionary innovations. Trends Genet..

[49] Wagner A (2012). The role of robustness in phenotypic adaptation and innovation. Proc. R. Soc. B Biol. Sci..

[50] Hayden EJ, Ferrada E, Wagner A (2011). Cryptic genetic variation promotes rapid evolutionary adaptation in an RNA enzyme. Nature.

[51] Wade MJ (1979). Sexual selection and variance in reproductive success. Am. Nat..

[52] Whitlock MC, Agrawal AF (2009). Purging the genome with sexual selection: Reducing mutation load through selection on males. Evolution (N. Y)..

[53] Sharp NP, Agrawal AF (2008). Mating density and the strenght of sexual selection against deleterious alleles in Drosophila melanogaster. Evolution (N. Y)..

[54] Sharp NP, Agrawal AF (2013). Male-biased fitness effects of spontaneous mutations in *Drosophila Melanogaster*. Evolution (N. Y)..

[55] Pischedda A, Chippindale A (2005). Sex, mutation and fitness: Asymmetric costs and routes to recovery through compensatory evolution. J. Evol. Biol..

[56] Arbuthnott D, Rundle HD (2012). Sexual selection is ineffectual or inhibits the purging of deleterious mutations in Drosophila melanogaster. Evolution (N. Y)..

[57] Westerman EL (2012). Biased learning affects mate choice in a butterfly. Proc. Natl. Acad. Sci..

[58] Joron M, Brakefield PM (2003). Captivity masks inbreeding effects on male mating success in butterflies. Nature.

[59] Morgan TH (1917). The theory of the gene. Am. Nat..

[60] Silber J (1980). Penetrance of the vestigial gene In *Drosophila melangoaster*. Genetica.

[61] Coyne JA, Prout T (1984). Restoration of mutationally supressed characters in Drosophila melanogaster. Heredity (Edinb)..

[62] Pezzoli C, Laporta D, Giorgi G, Guerra D, Cavicchi S (1986). Fitness components in a vestigial mutant strain of Drosophila melanogaster. Ital. J. Zool..

[63] Altenburg E, Muller HJ (1920). The genetic basis of truncate wing - An inconstant and modifiable character in Drosophila. Genetics.

[64] Brakefield PM, Kesbeke F, Koch PB (1998). The regulation of phenotypic plasticity of eyespots in the butterfly *Bicyclus anynana*. Am. Nat..

[65] Bacquet PMB (2015). Selection on male sex pheromone composition contributes to butterfly reproductive isolation. Proc. R. Soc. B Biol. Sci..

[66] R Development Core Team. R: A language and environment for statistical computing (2010).

[67] Bates D, Maechler M, Bolker B, Walker S (2015). Fitting linear mixed-effects models using lme4. J. Stat. Softw..

[68] Sokal, R. R. & Rohlf, F. J. *Biometry: the principles and practice of statistics in biological research* (Freeman, 1995).

[69] Nieberding, C. *et al*.. Partial restoration of mutational robustness after addition of genetic polymorphism and in the presence of sexual selection. *BioRxiv*, 10.1101/197194 (2017)

